# Literature Review of Omicron: A Grim Reality Amidst COVID-19

**DOI:** 10.3390/microorganisms10020451

**Published:** 2022-02-16

**Authors:** Suraj Arora, Vishakha Grover, Priyanka Saluja, Youssef Abdullah Algarni, Shahabe Abullais Saquib, Shaik Mohammed Asif, Kavita Batra, Mohammed Y. Alshahrani, Gotam Das, Rajni Jain, Anchal Ohri

**Affiliations:** 1 Department of Restorative Dental Sciences, College of Dentistry, King Khalid University, Abha 61321, Saudi Arabia; yalgarni@kku.edu.sa; 2 Department of Periodontology and Oral Implantology, Dr. H.S.J. Institute of Dental Sciences, Panjab University, Chandigarh 160014, India; vishakha_grover@rediffmail.com (V.G.); ohrianchal1993@gmail.com (A.O.); 3 Department of Conservative Dentistry and Endodontics, JCD Dental College, Sirsa 125055, India; 4 Department of Periodontics and Community Dental Sciences, College of Dentistry, King Khalid University, Abha 61321, Saudi Arabia; sshahabe@kku.edu.sa; 5 Department of Diagnostic Sciences and Oral Biology, College of Dentistry, King Khalid University, Abha 61321, Saudi Arabia; mshaik@kku.edu.sa; 6 Biomedical Statistician, Office of Research, Kirk Kerkorian School of Medicine at University of Nevada, 2040 W. Charleston Blvd., Las Vegas, NV 89102, USA; kavita.batra@unlv.edu; 7 Department of Clinical Laboratory Sciences, College of Applied Medical Sciences, King Khalid University, Abha 61321, Saudi Arabia; moyahya@kku.edu.sa; 8 Department of Prosthodontics, College of Dentistry, King Khalid University, Abha 61321, Saudi Arabia; 9 Department of Prosthodontics, Dr. H.S.J. Institute of Dental Sciences & Hospital, Punjab University, Chandigarh 160014, India; rajnijain25.rj@gmail.com

**Keywords:** Omicron, COVID-19, South Africa, B.1.1.529, cell-mediated immunity, vaccines, monoclonal antibodies

## Abstract

Coronavirus disease 2019 (COVID-19) first emerged in Wuhan city in December 2019, and became a grave global concern due to its highly infectious nature. The Severe Acute Respiratory Coronavirus-2, with its predecessors (i.e., MERS-CoV and SARS-CoV) belong to the family of *Coronaviridae*. Reportedly, COVID-19 has infected 344,710,576 people around the globe and killed nearly 5,598,511 persons in the short span of two years. On November 24, 2021, B.1.1.529 strain, later named Omicron, was classified as a Variant of Concern (VOC). SARS-CoV-2 has continuously undergone a series of unprecedented mutations and evolved to exhibit varying characteristics. These mutations have largely occurred in the spike (S) protein (site for antibody binding), which attribute high infectivity and transmissibility characteristics to the Omicron strain. Although many studies have attempted to understand this new challenge in the COVID-19 strains race, there is still a lot to be demystified. Therefore, the purpose of this review was to summarize the structural or virologic characteristics, burden, and epidemiology of the Omicron variant and its potential to evade the immune response.

## 1. Background

Coronavirus disease 2019 (COVID-19) first emerged in Wuhan city in December 2019, and became a grave global concern due to its highly infectious nature [1,2,3]. The Severe Acute Respiratory Coronavirus-2, with its predecessors (i.e., MERS-CoV and SARS-CoV) belong to the family of *Coronaviridae*. Reportedly, COVID-19 has infected 344,710,576 people around the globe and killed nearly 5,598,511 persons in the short span of two years [4]. As with other viruses, coronavirus constantly changes through genetic mutations, which have posed new challenges in the road to recovery. According to the United States (U.S.) government-led SARS-CoV-2 Interagency Group (SIG), SARS-CoV-2 variants can be categorized into four classes: Variant Being Monitored (VBM), Variant of Interest (VOI), Variant of Concern (VOC), and Variant of High Consequence (VOHC) [5]. Among the VOC class, variants such as Alpha (B.1.1.7), Beta (B.1.351), Gamma (P.1), and Delta (B.1.617.2 and AY lineages) evolved, and recently a new variant, B.1.1.529, was detected in several countries [5]. It quickly became a subject of discussion and exploration among the scientific community. The variant B.1.1.529 was first detected in Botswana, followed by South Africa between 11 November 2021 and 14 November 2021 [6]. It is a VOC due to its high transmissibility and less susceptibility to neutralization by antibodies produced either by previous viral exposure or vaccine administration [5,6]. According to the estimates, the cases of B.1.1.529 in South Africa grew by over fivefold in one week from 16 November to 25 November 2021 [7,8]. These upward trends remained for four weeks, followed by a rapid decline by 48% from 27 December 2021 to 2 January 2022 [7,8]. Other countries to report cases of infection by B.1.1.529 include, but are not limited to, France, The Netherlands, Germany, Portugal, Italy, the United Kingdom (U.K.), Canada, Hong Kong, Australia, and the United States. On 25 November, the B.1.1.529 variant was termed a Variant Under Monitoring by the United Kingdom Health Security Agency, and was considered as the maximally mutated variant amongst the other variants [7,8]. A day after, on 26 November 2021, this variant was designated as an official Variant of Concern by the World Health Organization (WHO) and named Omicron [9]. According to the epidemiological update provided by the European Surveillance System (TESSy), travel-related cases were identified in 13% of the confirmed infections, with nearly 70% of the remaining cases having arisen locally [10]. Omicron created chaos around the world and different studies are being conducted to study its presenting symptoms, transmission, risk of reinfection, severity, and its tendency to evade immune responses [11,12,13,14]. There are concerns related to its rampant transmission, which could hinder containment efforts, such as vaccine effectiveness. The surging trend in Omicron cases is worrisome, because this could cause an overwhelming demand on health care systems which have not yet completely recovered from the health and financial damages caused by the initial virus outbreak [14,15]. Although studies have attempted to understand this new challenge in the COVID-19 strains race, there is still a lot to be demystified. Therefore, the purpose of this review was to summarize the structural or virologic characteristics, burden, epidemiology of the Omicron variant and its potential to evade the immune response.

## 2. Structural Characteristics of the Omicron

The Omicron variant, which is a derivative of the Pango lineage B.1.1.529, exhibiting a variation in the 21 amino acid pertaining to the spike protein with the majority residing in the receptor binding domain (RBD) (residues 319–541) compared with the original strain [16,17,18,19,20,21]. SARS-CoV-2 has continuously undergone a series of unprecedented mutations and evolved to exhibit varying characteristics [16,17,18,19,20,21]. These mutations have largely occurred in the spike (S) protein (which is the site for antibody binding), which attribute high infectivity and transmissibility properties to Omicron variant [16,17,18,19,20]. According to the genomic reports, the S protein of Omicron has a total of 30 amino acid substitutions, 3 deletions, and 1 small insertion [21]. About 50% (n = 15) of amino acid substitutions occur exclusively in the receptor binding domain (RBD) [16,17,18,19,20,21]. Among the 15 RBD substitutes, N501Y and Q498R have a stronger affinity towards the angiotensin-converting enzyme (ACE-2 receptors), which explain the high transmissibility of the Omicron variant [16,17,18,19,20,21,22]. The ACE-2 receptors play a significant role in COVID-19 pathogenesis, which may involve serious organ failure [23].

## 3. Virologic Characteristics Explained (Omicron vs. Delta Variant)

Recent studies have reported that Omicron has a different mechanism for entering the host, and is capable of gaining cell entry independent of the transmembrane serine protease 2 (TMPRSS2) [24]. The entry pathway and viral replication of Omicron is through the endocytic pathway rather than TMPRS22 pathway, unlike Delta variant, which may have contributed to the differences in the disease presentation following exposure to Delta and Omicron variants [24]. TMPRS22 is highly expressed in alveolar cells of the lungs; thus, due to the lesser or no dependence of Omicron on the TMPRS22 pathway for its replication, lung involvement following exposure might be absent or limited [24,25]. In addition, the fusion capabilities to aid syncytia (structure that results following the fusion of multiple cells) formations are reduced in Omicron compared with the Delta variant. The reduced capacity of the syncytia formation translates to the lesser severity of the clinical manifestations and tissue tropism following the Omicron infection [24,25,26].

## 4. Epidemiology of Omicron and a Tropism Shift

Omicron spreads faster than the original virus; however, data related to its reproductive number remain limited. According to the European Center for Disease Prevention and Control, this variant could be more transmissible than the Delta variant [10]. Early reports from South Africa in November suggested the effective reproductive number (Re) of the variant to be in the range of 1.5–3; however, a recent estimate of Re as low as 0.75 was also reported [27]. These latest estimates should be interpreted with caution, because several factors, including changes in the testing measures or efforts and lag in case reporting, might have contributed to the change in the reproductive number [27]. The collective evidence suggests that Omicron has greater infectivity and potential to cause reinfection as compared with its predecessors [9,27]; however, data are insufficient to quantify its overall impact.

The symptoms of Omicron include a dry cough, scratchy throat, body aches, fatigue, runny nose, fever, and night symptoms [18,19]. According to recent evidence from South Africa, no peculiar symptoms associated with the variant have been reported, and some patients remained asymptomatic or only presented mild symptoms [10,20]. The patients infected with Omicron had fewer or no symptoms related to neurotropism (i.e., loss of taste and smell), which were peculiar with the earlier strains of the virus [18,28,29,30,31,32]. This tropism shift is attributed to the reduced dependence of Omicron on TMPRS22-expressing cells, such as the lower respiratory tract, brain, heart, kidney, and other extrapulmonary organs [33].

Among the initial Omicron VOC cases reported by the end of 2021 by EU/EEA countries to TESSy, 7 of every 10 cases were symptomatic [30]. Furthermore, the average age range for these reported cases was between 20 and 49 years [30]. The incidence of Omicron cases was higher among females compared with males [34]. The patients with preexisting conditions and those with the acute respiratory attacks were among the Omicron-associated hospitalizations [34]. Furthermore, it was reported that the risk of hospital and ICU admissions was lower compared with the preceding waves in South Africa [34]. In addition, a lower proportion of patients required oxygen therapy and mechanical ventilation [34]. The median length of stay was reduced by half in this current wave as compared with what was observed in the previous waves (3–4 days vs. 7–8 days) [34]. Preliminary data from South Africa suggested that Omicron causes less severe symptoms than the original SARS-CoV-2 virus, although rapidly increasing case loads and overall transmissibility remain the serious concerns. Omicron seems to weaken the association between cases and mortality, which is supported by the U.K.-based evidence, according to which the Omicron case rate in the United Kingdom was 35% per day; however, the death rate continuously declined, unlike the patterns which were observed in the first wave of the COVID-19 pandemic [30,35]. Data also suggested that every 3 in 10 cases which emerged in the existing cases were fully vaccinated, which points to the ability of Omicron to evade immune response [34].

## 5. Potential for Immune Escape

The population-level evidence from South Africa estimated that the hazard ratio for Omicron reinfection and primary infection was 2.39, suggestive of a possible evasion from natural immunity gained from previous infections [12,36]. This is consistent with the general premise that antibodies generated following the natural response have a lower titer and greater dissipation, thereby reducing immunity over time [12,36]. It is important to explore the dimensions of vaccine effectiveness, especially between countries which have used different types of vaccines. Given the genetic divergence of Omicron with its predecessors, its emergent genetic sequence coding the spike protein has been documented to sheath away from the respective immunoglobulins or humoral response [37]. Upon analyzing the titers of neutralizing antibodies of sera from vaccinated individuals, the neutralization capacity was lower for Omicron [37,38,39,40,41,42,43]. However, the neutralization capacity was maintained among vaccinated individuals, who also had a history of prior infection [38,39]. Interestingly, one study by Cele et al. [44] reported high levels of neutralizing antibodies in the plasma of subjects who had a history of previous infection and were fully vaccinated. According to an (unpublished) report by Andrew et al., the vaccine effectiveness was raised by 75–80% following the administration of a booster dose [45]. This might be relatively lower than the Delta variant, but still encouraging to deal with the unknowns of Omicron. Some studies have compared the immunogenic properties of homologous and heterologous vaccinations, and found that the heterologous prime-boost regimens exhibited higher neutralizing activity compared with the homologous vaccinations [46]. More data will be needed to confirm this finding. Existing data suggest a reduced efficiency of vaccines in antibody neutralization; however, the cell-mediated immunity remains resilient in offering protection from severe illness [37,38,39,40,41,42,43,47]. T cells offer cell-mediated immunity and have an ability to recognize mutated virus through multiple sites beyond the spike protein [37,38,39,40,41,42,43,44,45,46,47]. In addition, T cells provide long-lasting immunity which does not fades as quickly as natural antibodies [45,46,47,48].

## 6. Laboratory Testing

Although reverse transcription—polymerase chain reaction (RT-PCR) tests remain the gold standard in COVID-19 testing, many rapid antigen detection tests (RADTs) and self-RADTs have been introduced and widely adopted. With the emergence of new variants, reductions in the RADT sensitivity have not yet been reported [49]. Studies have reported the effectiveness of antigen tests in detecting the Omicron variant at the lowest dilutions [49]. It is important to note that studies which will provide more insight to the analytical sensitivity of RADTs to detect the Omicron variant in settings with high transmission are ongoing. RT-PCR-based S-gene target failure (SGTF) assays that do not detect the S-gene with Δ69–70 deletion may serve to screen for the Omicron VOC [49,50]. Screening for VOC-specific amino acid substitutions can also be performed using specific RT-PCR assays targeting single-nucleotide polymorphisms (SNPs) [49]. Some commercially available SNP test kits for the identification of T478K, N501Y and P681H fail to reliably identify these mutations for Omicron, even with S-gene mutations [50]. The U.S. FDA has identified and listed molecular tests that may be impacted by new Omicron variant mutations [51]. The currently widely used real-time RT-PCR tests can detect the new variant, but rapid antigen detection tests may need more confirmatory studies in this regard.

## 7. Therapeutics

The WHO established its Joint Advisory Group on COVID-19 with the aim of collecting and synthesizing evidence related to the potential impact of the emergence of Omicron on the effectiveness of drugs currently being used or under investigation [52,53,54]. At present, limited evidence is available regarding the effectiveness of bioequivalents of monoclonal antibody therapies, including Sotrovimab, Casirivimab, Imdevimab, Bamlanivimab, etc. [54] against Omicron. Some early reports have indicated that the cocktail mixture of Casirivimab and Imdevimab does not neutralize Omicron in vitro [53], whereas the neutralization capability of Sotrovimab was retained against Omicron [53,54]. There is presumptive thought based on the genetic analysis regarding the efficacy of Remedisivir that it may continue to be active against Omicron; however, laboratory confirmative study data are needed [53,54]. Clinical and laboratory data on the effectiveness of newer oral antivirals against Omicron are not yet available. Owing to genomic changes, the variant might be responsive to some available treatments and may not respond to other modalities effective against the original virus. To assess the effect on the three currently available monoclonal antibody treatments (Sotrovimab, Bamlanivimab and Etesevimab, and REGEN-COV) more data need to be evaluated [54].

## 8. Public Health Response to Minimize Omicron Transmission

Similarly to other infectious and highly transmissible diseases, efforts at macro (country level) and micro level (individual level) are vital. First and foremost, surveillance plays an integral role in the public health response in curbing the transmission of Omicron. Recently, the CDC established a genomic surveillance system to improve the understanding of predominant strains circulating in the community. The CDC has partnered with commercial laboratories and universities to supplement public health sequencing efforts and conduct genomic surveillance research [55]. According to the WHO recommendations, countries should extend their research infrastructure to develop a science-based approach to curb the spread of COVID-19. Ensuring equitable access to healthcare will be critical to overcoming the disparate effects of the pandemic and new challenges induced by the mutated strains. At individual level, physical distancing, the use of masks, avoiding public gatherings and getting vaccinated will remain useful in curbing the spread.
